# Cross-cultural adaptation of the Brazilian version of the Athlete Sleep Behavior Questionnaire

**DOI:** 10.5935/1984-0063.20200105

**Published:** 2021

**Authors:** Lucas Alves Facundo, Maicon Rodrigues Albuquerque, Andrea Maculano Esteves, Matthew W Driller, Isadora Grade, Marco Túlio De-Mello, Andressa Silva

**Affiliations:** 1 Universidade Federal de Minas Gerais, Departamento de Esportes - Belo Horizonte - Minas Gerais - Brazil.; 2 Centro de Treinamento Esportivo - CTE, Universidade Federal de Minas Gerais, Departamento de Esportes - Belo Horizonte - Minas Gerais - Brazil.; 3 Faculdade de Ciências Aplicadas - Universidade Estadual de Campinas, Ciências Aplicadas - Limeira - São Paulo - Brazil.; 4 Sport and Exercise Science, School of Allied Health, Human Services and Sport - Melbourne - Victoria - Australia.

**Keywords:** Circadian Rhythm, Sports, Sleep Hygiene, Sleep Disorders, Surveys and Questionnaires, Behavior Rating Scale

## Abstract

**Introduction:**

Considering the specificity of the sporting context and the influence of sleep on athletic performance, the “athlete sleep behavior questionnare” (ASBQ) was developed to evaluate sleep behavior in English-speaking athletes.

**Objective:**

The aim of this study was to perform a cross-cultural adaptation and validation of the ASBQ in Brazilian athletes.

**Methods:**

The cross-cultural adaptation was processed by procedures of translation and back-translation. Content validity was performed by 9 experts, calculating the coefficient of content validity for the equivalence of the individual items (Cvci) and the total global score (Cvct), in addition we conducted a pilot study using the translated version of the questionnaire. The next phase of the study included 52 athletes completing the translated ASBQ. The reliability of the questionnaire was assessed by the intraclass correlation coefficient (ICC), standard error of measurement (SEM) and by Cronbach’s alpha (α).

**Results:**

The ASBQ passed the process of cross-cultural adaptation, obtaining the Brazilian Version of the ASBQ (ASBQ-BR), and with acceptable values of Cvci (0.89-1.00) and Cvct (0.96). Additionally, the ASBQ-BR showed acceptable values of reliability (ICC=0.857; Cronbach’s α=0.78) and a SEM of 3.05 AU.

**Conclusion:**

The ASBQ was translated to a newly developed ASBQ-BR, resulting in acceptable values for content validity and reliability. The ASBQ-BR provides a valuable tool for monitoring sleep behaviors in Brazilian athletes.

## INTRODUCTION

Sleep is an important process that can both impair and improve the health of humans and animals^1,2^. Sleep plays a vital role in sensorimotor and athletic performance, providing physical, coordinative, and cognitive recovery^3,4^. Sleep with adequate duration and quality, can optimize sports performance and reduce the likelihood of musculoskeletal injuries in elite athletes^5,6^. However, it has been reported that many athletes do not obtain enough sleep and may exhibit sleep disturbances^7^. In a study by Lastella et al. (2015)^8^, results demonstrated that athletes had an average of 6.8±1.1 hours of sleep per night, a duration that doesn’t meet the recommended duration for this population of 9-10 hours per night^8,9^. Additionally, in the study of Silva et al. (2019)^7^, results highlighted that Olympic athletes sleep an average of 5.5 hours per night, and 53% of Brazilian Olympic athletes (n=146) reported complaints relating to sleep, including ~36% displaying sleep disorders, with insomnia being the main complaint^7^. Disturbed sleep quality may impair physical recovery and subsequent sports performance^10^.

There are many specific factors in the athletic context that can negatively impact on athlete recovery^11^. This population regularly faces long and short-haul travel for competition, night matches/competition, and a high frequency of caffeine use as well as other stimulants^11^. Further, in the days before competition, it is common for athletes to exhibit nervousness and anxiety, making it harder to fall asleep^12^. Over time, these complaints of anxiety, stress, schedule of competitions, and a non-restorative sleep could have an impact on performance^13^. Concomitantly, the common scheduling of training in the morning may further impair the time available for sleep^14^. A wide array of interventions can compensate in these situations, for example, sleep hygiene education^11^ and sleep extension have been explored with positive findings in some studies^15,16^. However, for appropriate interventions to be implemented, precise tools are required to first obtain information on the athlete’s current sleep habits and behaviors.

Different tools are used for the monitoring of sleep in athletic populations. Actigraphy is often used for sleep/wake cycle analyses^17^, where the analysis of sleep phases patterns and sleep disturbances is performed using polysomnography, the gold standard for sleep evaluation^18^. Another important way to evaluate sleep of athletes is the use of questionnaires that are more cost and time effective. Also, they can evaluate a wide range of sleep measures and behaviors^17^. Additionally, questionnaires, scales and surveys are very common to analyze the sleep of athletes, in team and individual sports^18,19^. New sleep questionnaires have recently emerged that are specific to the sport and athlete setting^17^.

Brazil is a leader in the world when it comes to athlete-sleep research. A recent study reported that it is host to one of the most productive institutions in the world, and is also in the top 10 as a country when it comes to athlete-sleep research (20). Different tools have been used by Brazilian researchers to evaluate aspects related to the sleep-wake cycle. Relating to questionnaires, few options are available to practitioners working with Brazilian athletes, for example, the Pittsburgh sleep quality index (PSQI) and the Epworth sleepiness scale (ESS) have both been validated in Brazilian Populations^21,22^ and used in studies with elite athletes^23^. Another common tool includes the use of sleep diaries, where data can be collected over long time-frames and allow for circadian rhythm analysis in a reliable way^24^. Nevertheless, these questionnaires (PSQI and ESS) are not specific to athletes, as they do not evaluate particular aspects and challenges faced by this population and can impact their results^25^. This is the main point that highlight the relevance of adapt questionnaires specific for Brazilian athletes.

One of the specific questionnaires developed for athletes is the ATHLETE SLEEP BEHAVIOR QUESTIONNAIRE (ASBQ)^26^. The ASBQ evaluates information related to the practices of sleep behaviors of elite athletes, making it possible for individualized feedback and interventions based on the answers provided on 18 specific items. However, this questionnaire was developed for English-speaking athletes and has recently been translated and validated for Turkish athletes^27^, but is yet to be translated and validated for Brazilian athletes. Therefore, the aim of the current study was to ensure the cross-cultural adaptation, translation and validation of the ASBQ for Brazilian athletes, in the Portuguese language. Additionally, we aimed to test the reliability of the translated version.

## MATERIAL AND METHODS

### Ethical approval

The present study was approved by the research ethics committee at the Federal University of Minas Gerais (N. 01912318.6.0000.5149 and N. 64492016.8.0000.5149).

### Participants

This study included a convenience sample of 52 athletes.

Group 1: athletes (n=52), average age of 27±8 years (male=41; female=11), from the sports of: athletics (n=7), taekwondo (n=8), swimming (n=7), judo (n=4), triathlon (n=6), Olympic weightlifting (n=1), para weightlifting (n=8), and volleyball (n=11); 15 were para-athletes with a disability.

As the inclusion criteria for athletes, we included those who competed at National or International level events over the past last year, and were >18 years of age at the time of completing the study. The data related to the ASBQ was collected in person, with the main researcher present to answer any questions of the individuals, and during the competitive season of their sport.

### Cross-cultural adaptation

**Figure 1:**
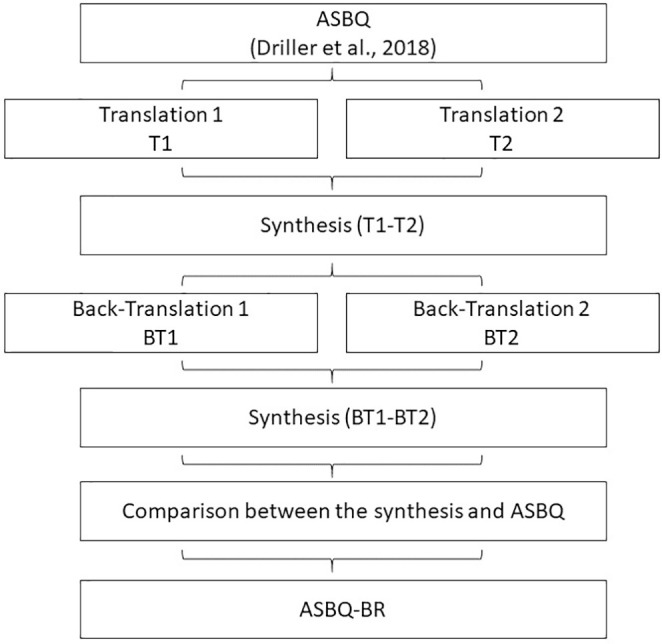
Procedures of cross-cultural adaptation.

The process of translating the questionnaire followed the steps proposed by Guillemin et al. (1993)^28^ and Beaton et al. (2000)^29^. Figure 1 shows these procedures in a schematic manner.

### Step 1

Two translators performed independent translation of the questionnaire from the English language to the Portuguese language. Both translators are fluent in English and one of them has experience in the sporting context. After the conclusion of translation 1 (T1) and translation 2 (T2), the researcher met with the two translators and with two other experienced researchers in the process of questionnaire adaptations and with sleep research, to compare the two versions of the translations and evaluate if there were any discrepancies. After this process, a preliminary version (T1-T2) was developed through the synthesis of the translations.

### Step 2

The synthesis obtained previously by the two translators (T1-T2) was reverse translated to the English language. The process was performed by two independent translators, both of them with English as their native language and fluency in the Portuguese language. Again, the researcher met with the translators, with the two independent versions: back-translation 1 (BT1) and back-translation 2 (BT2) and built a second version with the combination of these two versions (BT1-BT2).

### Step 3

In the next step, the different versions of the questionnaire, T1-T2 and BT1-BT2, were compared with the original version of the ASBQ. In this meeting the final version (ASBQ-BR) of the questionnaire was decided on by the main researcher and the translators involved.

### Step 4

Semantic and syntactic verification of the last version was performed. The questionnaire was reviewed by a professional specialized in grammatical review of texts in the Portuguese language. In addition, a pilot study was conducted with 27 athletes aiming to improve the understanding of the questionnaire items. The volunteers were athletics’ junior athletes with a medium age of 17±2 years old. The volunteers were asked to point out issues with difficulties in interpretation/understanding. These answers in the ASBQ were not used in the psychometric analyses.

In the process of direct translation and reverse translation, four qualified translators were recruited. In the first step, one of the translators had a Master’s degree in sports sciences and certification in the English language, with international experience in the sports field, in addition to writing scientific articles in English. Additionally, the other professional had certification in the English language with no experience in the sports context.

In the second step, both translators were of North American origin, more precisely from the United States, and had lived in Brazil for more than 7 years and have fluency on Portuguese. In addition, one of the translators was an English teacher and professional translator of academic texts living and working for more than 10 years in Brazil.

### Content validity

Content validity is related to the extent to which the items of a given instrument are associated with the construct that will be measured, in this case, the sleep behavior of athletes^30^. In this process, a qualitative approach was used by specialists in the field. These experts assess the relevance of each item to the construct analyzed by the questionnaire. The validity can be calculated by the content validity coefficient (CVC) per individual item (Cvci) and by the total content validity coefficient (Cvct)^31^.

To calculate the content validity coefficient for each item and the total score, the steps proposed by Hernández-Nieto (2002)^31^ were performed. In addition, in the form, there was a space for professionals to make any suggestions.

### Procedures

The researchers implemented the content validity online using the Google Forms platform. According to the scientific literature, a minimum number of 4 to 6 experts are recommended to perform the content validity, and it is not necessary to exceed the number of 10 evaluators^32^. Nine professionals were recruited, considered experts in the sleep area and with experience in applying these concepts in the context of athletes. These evaluators have a Ph.D. or Master degree as well as practical and academic experience in the field of sleep and athletes, and included medical physicians, physical education professionals, physiotherapists, psychologists, and biologists. In the form sent to the researchers, the objectives and the importance of the procedures were explained. They were then asked to assess the equivalence of each item in the questionnaire for the athletes’ sleep behavior construct. The participants answered a scale with a score from 1 to 4 for each item, being: 1 = non-equivalent item; 2 = item needs major revision to assess equivalence; 3 = equivalent item, needs minor changes; 4 = absolutely equivalent item. These items were used based on the suggestion of Souza et al. (2017)^33^.

## RELIABILITY

### Instruments

#### Athlete’s sleep behavior questionnaire

The ASBQ was developed using the combination of the sleep hygiene index, the International Classification of Sleep Disorders, and studies that described the most common issues related to athletes’ sleep^12,34^ together with the recommendations related to these specific issues^9,35,36^. The ASBQ is an 18-item questionnaire that includes questions related to habits considered of concern to athletes in their sleep hygiene and related to their behavior in relation to sleep. This tool was created in order to identify areas in which it is possible to improve the athletes’ behavior in relation to their sleep hygiene and to screen their habits regarding this aspect.

The questionnaire presents as a total global score, the sum of the answers in the questions. Responses related to the frequency of specific behaviors are scored as follows: 1 = never, 2 = rarely, 3 = sometimes, 4 = often, and 5 = always. These questions are distributed in 3 factors of the questionnaire: routine and environmental factors (items 1, 5, 15, 16, 17, and 18); behavioral factors (items 2, 4, 8, 10, 11, 12, and 13); and factors related to sport (items 3, 6, 7, 9, and 14). The researchers that developed this questionnaire suggest that scores less than or equal to 36 AU indicate “good sleep behavior” and above 42 AU represent “poor sleep behavior”^26^. Scores between 36 and 42 AU classify the athlete with “moderate sleep behavior”. Good sleep behavior (≤36) represents an average “rarely” response in the 18 items, while the upper threshold, or poor sleep behavior (≥42), requires more than one answer for items such as “sometimes”, “frequently” or “always”^26^.

#### Test-retest reliability and internal consistency

The reliability of an instrument refers to the ability of an instrument to reproduce a result consistently (test-retest reliability or stability), in addition to the domains of an instrument that measure the same characteristic (internal consistency)^33^.

The test-retest reliability of the questionnaire was performed by administering the questionnaire on two different occasions for the same individual^30^. In the present study, the time used to assess the test-retest reliability was seven days between applications. A cohort of 34 athletes was used to assess reliability. This time between tests was the same as used previously in the construction of the English ASBQ^26^ and also used in the Turkish version of the questionnaire^27^.

#### Statistical analysis

To analyze the test-retest reliability, the intraclass correlation coefficient (ICC) was used. The type of ICC used was the ICC (2,k), based on a mean-rating (k=2), absolute agreement, 2 way random-effect^37^.To guarantee the reliability of the instrument, values above 0.7 were considered acceptable^38^. Moreover, the standard error of measurement (SEM) was used. To calculate the SEM we used the “rel” R package^39^.

Regarding the analysis of internal consistency, we opted to use Cronbach’s alpha. Cronbach’s alpha coefficient assesses the average of intercorrelations between items within a scale^30^. Studies generally use a minimum threshold Cronbach’s alpha value of >0.7 as recommended, but values between 0.6 and 0.7 can be interpreted as satisfactory^38^.

Calculations of CVC and effect size were performed in Microsoft Excel. For the calculation of ICC and SEM, R Statistics software was used, while for Cronbach’s α, SPSS version 19 was used.

## RESULTS

### Cross-cultural adaptation

In the pilot study, the athletes reported that the items of questionnaire were clear and did not show any issues. The score of these athletes was 36±5 AU on the ASBQ-BR. The translation result can be seen in Table 1

**Table 1:** Result of the cross-cultural adaptation.

Original English version (ASBQ)	Portuguese version (ASBQ-BR)
I take afternoon naps lasting two or more hours	Tirei cochilos à tarde que duraram duas ou mais horas
I use stimulants when I train/compete (e.g., caffeine)	Utilizei estimulantes para treinar/competir (ex.: cafeína)
I exercise (train or compete) late at night (after 7 p.m.)	Exercitei-me (treinei ou competi) tarde da noite (após 19 horas)
I consume alcohol within 4 hours of going to bed	Consumi álcool no período de até 4 horas antes de ir me deitar
I go to bed at different times each night (more than ±1 hour variation)	Deitei-me em horários diferentes a cada noite (mais de uma hora de variação)
I go to bed feeling thirsty	Deitei-me sentindo sede
I go to bed with sore muscles	Deitei-me com dores musculares
I use light-emitting technology in the hour leading up to bedtime (e.g., laptop, phone, television, video games)	Utilizei tecnologia que emite luz na hora que antecede o momento de ir me deitar (ex.: computador, celular, televisão, videogames)
I think, plan and worry about my sporting performance when I am in bed	Pensei, planejei e/ou me preocupei com meu desempenho esportivo quando estava deitado para dormir
I think, plan and worry about issues not related to my sport when I am in bed	Pensei, planejei e/ou me preocupei com questões não relacionadas ao meu esporte quando estava deitado para dormir
I use sleeping pills/tablets to help me sleep	Utilizei medicamentos para me ajudar a dormir
I wake to go to the bathroom more than once per night	Acordei para ir ao banheiro mais de uma vez por noite
I wake myself and/or my bed partner with my snoring	Acordei e/ou acordei meu companheiro de cama com meu ronco
I wake myself and/or my bed partner with my muscle twitching	Acordei e/ou acordei meu companheiro de cama com movimentos involuntários
I get up at different times each morning (more than ±1 hour variation)	Levantei-me em horários diferentes cada manhã (mais de uma hora de variação)
At home, I sleep in a less than ideal environment (e.g., too light, too noisy, uncomfortable bed/pillow, too hot/cold)	Em casa, eu dormi em um ambiente não ideal para o sono (muito claro, muito barulhento, em cama e/ou em travesseiro desconfortável, muito quente/frio)
I sleep in foreign environments (e.g., hotel rooms)	Dormi em ambientes desconhecidos (ex.: quartos de hotéis)
Travel gets in the way of building a consistent sleep-wake routine	Viagens me atrapalharam a seguir uma rotina consistente de dormir e acordar

Source: The authors.

### Content validity

All items presented acceptable values (>0.8) in relation to the equivalence for the construct of the athlete’s sleep behavior (Cvci). The item values ranged between 0.89-1.00. In addition, the Cvct presented an acceptable score for the total content validity, with a value of 0.96 (Table 2).

**Table 2:** Content validity coefficient.

Item	Cvci
1	0.89
2	0.92
3	0.92
4	0.94
5	0.97
6	0.97
7	1.00
8	0.94
9	0.97
10	0.94
11	1.00
12	0.97
13	0.94
14	0.94
15	0.97
16	0.97
17	0.94
18	0.97
Cvct	0.96

Source: The authors.

### Reliability

The ICC resulted in acceptable scores of 0.857 and with a 95% confidence interval of 0.734-0.926 [F(33.34)=13.1, *p*=0.001]. The statistical power of this analysis was 1, which is considered high. Additionally, the average difference in the total global score between the two applications was 0.9±4.0 AU. Another metric used was the SEM that shows a value of 3.05 AU (2.35- 3.74 AU 95% CI). The variation between the first and the second application is inside of the SEM. In other words, it represents that the difference between the two applications, is not a real score difference. In the analysis of internal consistency, a Cronbach’s α of 0.78 was found with a 95% confidence interval.

## DISCUSSION

The aim of the present study was to perform a cross-cultural adaptation, translation and validation of the English version of the ASBQ to the Portuguese language. The ASBQ was translated following the steps proposed in the scientific literature^29^, and demonstrated acceptable levels of validity in relation to the athletes’ sleep behaviors. The ASBQ-BR also presented acceptable levels of reliability.

Adequate sleep is essential for not only sports performance, but also in the regulation of almost all body systems, ensuring hormonal, cardiac, respiratory, and immunological performance and balance^40^. However, not all athletes manage to obtain the necessary amount of sleep, possibly related to having inadequate sleep hygiene habits. This supports the need to monitor and control these behaviors to enhance athletic performance and prevent injuries^40,41^. From this perspective, the development of specific tools to monitor the sleep-wake patters of athletes is vital^25^.

Different tools are used to monitor the sleep of elite athletes, from high-cost tools, such as actigraphy and polysomnography, to less expensive instruments such as mobile applications and validated questionnaires^17^. The use of questionnaires, in addition to the low cost, has the advantage of collecting behavioral data, such as sleep habits and practices^42^. Additionally, questionnaires are one of the most commons tools used to athletes’ sleep monitoring^18,19^. Thus, questionnaires may provide us with valuable information about the sleep and recovery practices of athletes, guiding precise and effective intervention protocols and strategies for this population^43^. Therefore, the importance of the adaptation and translation of questionnaires to native languages within the scientific, practical and clinical setting is imperative.

In the current study, the ASBQ underwent a cross-cultural adaptation procedure to obtain ASBQ-BR. In the process of translating the questionnaire, we believe that the presence of two independent translators with different experiences (one from the sports environment and the other outside this context) positively influenced this process. While the translator with experience in the sports field guaranteed a more contextualized translation, the translator who did not have these characteristics was able to highlight ambiguities in the original questionnaire related to language, without interference from the conceptual sports bias^29^. In summary, the experience and qualification of the working group involved in the translation process can indicate quality in the process^44^.

In the translation of the ASBQ to the ASBQ-BR version, the translation and reverse translation procedures were performed. Although there is still no gold standard for the translation of scales and questionnaires, the reverse translation procedure is indicative of a robust process for cross-cultural and language translation studies, as it guarantees the semantic and syntactic maintenance^44^. Furthermore, to ensure comprehension and grammatical correction, a thorough review and a pilot study were carried out. After this procedure, the clarity of the ASBQ-BR questions was ensured^44^.

Another important step in the process of cross-cultural adaptation of the questionnaire is content validation, which represents how closely the items in the questionnaire are related to the evaluated construct^30^. All items in the questionnaire had appropriate values ranging from 0.89 to 1.00 (Cvci), in addition to the total assessment of the questionnaire (Cvct) presenting an appropriate score of 0.96^45^. In this procedure, the equivalence of each item was assessed in relation to the athletes’ sleep behavior construct^46^. In this way, through the evaluation of specialists, the equivalence of the items in this questionnaire was confirmed in the evaluation of behaviors that influence the sleep effectiveness of athletes.

The evaluation of the test-retest reliability of the ASBQ-BR ensures the stability of the tool by evaluating the same situations or phenomena at different time-points^47^. In other words, reliability refers to the consistency of the measurement, in which can be assessed by absolute and relative consistency^48^. Absolute consistency concerns the consistency of scores of individuals, and the SEM is a statistical method used to this purpose^48^. On the other hand, relative consistency refers to the consistency of the position to others, and can be quantified using the ICC^48^. In summary, our results showed acceptable levels of reliability. There was a difference in the total global score of only 0.85 AU between the first application and the second application 7 days later, and the ICC presented a value of 0.857. In the original English version of the ASBQ validation study, a value of 0.87 was found for the ICC, in addition to a difference of 0.10 AU between applications^26^. Similarly, in the study where the ASBQ was translated in the Turkish language, acceptable values of 0.85 were also found^27^. The result of the SEM reported that a true value would vary between 3.05 A.U. The mean difference between the two applications of the questionnaire of 0.9, show us that the results of the ASBQ-BR, are in a range of true results^48^. In addition, it will only be possible to check difference between the two applications of the questionnaire when the difference is greater than SEM=3.05. Thus, high scores on test-retest reliability demonstrate that the ASBQ-BR is stable during two applications and provide positive indications for it use in the practical and scientific field^26^.

The ASBQ-BR reported an acceptable value for Cronbach’s alpha, which was 0.78. This value was higher than those found in the original ASBQ development study (0.63) and also in the Turkish language study (0.62)^26,27^. The Cronbach’s alpha test is important to use for questionnaires that analyze different behaviors of a person and their personality^49^. It is important to note that an acceptable value of reliability represents that the items evaluate the same construct that they are related to, in this case, the sleep behaviors of athletes^30^.

Another relevant finding in the present study was the mean ASBQ-BR score of 43.81 AU. Following the criteria established by Driller et al. (2018)^26^ in creating the ASBQ, this score can be characterized as “poor sleep behavior”, which also occurred within the sample of the original study^26^. These findings may demonstrate that, on average, Brazilian athletes in our sampled population show poor sleep behavior. From this perspective, it is important to emphasize the need for constant monitoring of sleep hygiene habits of Brazilian athletes, with specific tools for this population, in order to propose necessary interventions for their improvement^17^. Additionally, the ASBQ has been shown previously to be effective in improving these habits, paying attention to the individual items on the questionnaire in which the athlete has a higher frequency reported, and targeting approaches and interventions to improve these behaviors^43^. Improving behaviors as the sleep duration can be important for the maintenance of the cognitive and athletic performance^2,50^.

The ASBQ-BR is the first questionnaire for analyze the specific sleep behavior of athletes in Brazil as we know. The ASBQ-BR will help the work of coaches, researchers and athletes to analyze sleep hygiene. Additionally, no differences were found in the scores between the sexes (Mann-Whitney U=225.00; Z=-0.011; *p*=0.991), and in volunteers with disabilities and without physical disabilities (Mann-Whitney U=198.00; Z=-1.609; *p*=0.108), making this questionnaire suitable for different competitive contexts.

In conclusion, the ASBQ went through thorough and robust methods in the process of cross-cultural adaptation and translation to Brazilian Portuguese, obtaining new validation of the Brazilian version of this questionnaire, the ASBQ-BR. Additionally, the equivalence of its items in relation to the sleep behavior of athletes was also verified (Cvci=0.89-1.00; Cvct=0.96), as well as its reliability (ICC=0.857; Cronbach’s α =0.78) in a 95% confidence interval. The ASBQ-BR may provide a valid, reliable and valuable tool for researchers, practitioners or clinicians working with Brazilian athletes looking to target maladaptive sleeping behaviors.
